# Longitudinal Course of Long Finger Flexor Shortening in Males with Duchenne Muscular Dystrophy: A Retrospective Review^1^

**DOI:** 10.3233/JND-221653

**Published:** 2024-01-02

**Authors:** Saskia L.S. Houwen-van Opstal, Menno van der Holst, Michel A.A.P. Willemsen, Erik H. Niks, Imelda. J.M. De Groot, Edith H.C. Cup

**Affiliations:** a Department of Rehabilitation, Amalia Children’s Hospital, Radboud University Medical Center, Nijmegen, The Netherlands ORCID: 0000-0002-9221-5679; b Department of Orthopaedics, Rehabilitation and Physiotherapy, Leiden University Medical Center, The Netherlands ORCID: 0000-0002-0797-5711; c Donders Centre for Neuroscience, Department of Pediatric Neurology, Amalia Children’s Hospital, Radboud University Medical Center, Nijmegen, The Netherlands ORCID: 0000-0001-7860-7791; d Department of Pediatric Neurology, Leiden University Medical Center, The Netherlands ORCID: 0000-0001-5892-5143; e Donders Centre for Neuroscience, Department of Rehabilitation, Amalia Children’s Hospital, Radboud University Medical Center, Nijmegen, The Netherlands ORCID: 0000-0003-1634-1427; f Donders Centre for Neuroscience, Department of Rehabilitation, Radboud University Medical Center, Nijmegen, The Netherlands ORCID: 0000-0003-3452-9650

**Keywords:** Duchenne muscular dystrophy, long finger flexors, goniometry, brooke score, longitudinal course

## Abstract

**BACKGROUND:**

Shortening of the long finger flexors (Flexor Digitorum Profundus, FDPs) in Duchenne Muscular Dystrophy (DMD) causes reduced hand function. Until now, longitudinal studies on the natural course of the shortening of the FDPs are lacking, which impedes recommendations on timing and evaluation of preventive measures.

**OBJECTIVE:**

To investigate the longitudinal course of the FDP length during different disease stages focusing on symmetry, timing, and decline of the FDP length.

**METHODS:**

A retrospective, longitudinal multicenter study was conducted in the Radboud university medical center and the Leiden university medical center. The FDP outcome was measured using goniometry and gross motor function was assessed using the Brooke score. Longitudinal mixed model analyses were used to describe the course of the FDP outcome, and to investigate symmetry in both hands.

**RESULTS:**

Data on 534 visits of 197 males (age ranged 4–48 years) showed that in the ambulatory stages the FDP outcome was within a normal range. The mean decline in FDP outcome is 3.5 degrees per year, the biggest decline was seen in Brooke 5 (>15 degrees per year). In Brooke 4, 41% of the FDP outcome was < 40 degrees. No significant differences were found between right and left.

**CONCLUSIONS:**

This study supports the consideration of preventive measures to delay shortening of the FDPs in DMD patients transitioning to a Brooke scale of 4 or higher. Besides, natural history of FDP outcome has been established, which provides a base to evaluate (preventive) interventions.

## 1 INTRODUCTION

Duchenne muscular Dystrophy (DMD) is an x-linked, severe progressive muscular dystrophy affecting 15.9 to 19.5 per 100.000 live births [1, 2]. DMD typically comprises of proximal muscle weakness in the early stages of the disease, leading to loss of ambulation around 8–14 years, and more distal weakness, also affecting arm and hand function in the later disease stages [3]. Due to the introduction of mechanical ventilation, scoliosis surgery, corticosteroids and the improvement of multidisciplinary care, survival beyond twenty years is common [4].

The extended life expectancy provides more opportunities for education, work and other activities, which continue to demand arm- and hand function. However, arm function decreases due to progressive muscle weakening in people with DMD and with this also the ability to perform activities with the upper extremity (UE) [3]. Besides decrease of muscle strength, muscles shortening is common in DMD. The typical pattern of upper extremity muscles shortening in DMD includes decreased supination, ulnar deviation and shortening of the long finger flexors (flexors digitorum profundus, FDPs) [5]. FDP shortening results in decreased ability to extend the wrist with extended fingers, which is crucial in positioning hands during activities, for example with typing [6].

In current clinical practice, hand orthoses are advised to delay FDP shortening. However, evidence for effectiveness of these orthoses is limited. Weichbrodt et al. [7] studied 8 people with DMD with passive wrist extension less than 50 degrees (with extended fingers), and found that hand orthoses could delay development of contractures. In general, a wrist extension of 40 degrees is needed for performance of precision tasks [8]. From our clinical experience and previous research [9], we know that the compliance of wearing hand orthoses is limited; people with DMD already have extensive care rituals, and orthoses can cause discomfort and further limit the functionality. On the other hand, we found that the participants were motivated to preserve their hand function and a personalized wearing schedule was helpful [9]. Currently knowledge on the course of FDP shortening during the different disease stages (i.e. early ambulant, late ambulant, early non-ambulant, late non-ambulant) is lacking [10]. More insight in the course of the FDP shortening is needed to decide on the most appropriate timing of interventions and to be able to evaluate the effect of preventive measures such as hand orthoses on the delay of FDP shortening.

We aim to investigate the longitudinal course of the FDP shortening during different disease stages in both hands, focusing on timing, symmetry and decline of the length of the FDPs.

## 2 METHODS

### 2.1 Data collection

A retrospective longitudinal multicenter study was carried out using clinical data registered in the Dutch Dystrophinopathy Database (DDD). Data was collected between January 2014 and March 2022 in the two national reference centers in the Netherlands, Radboud university medical center and Leiden university medical center. Both centers are part of the Duchenne Center Netherlands. Within this collaboration outcome measures are aligned and neuromuscular therapists are jointly trained in the use of these outcome measures. Parameters were derived as part of the standards of care during annual visits to the outpatient clinics where patients were assessed by trained physiotherapists and occupational therapists. Inclusion criteria were: the DMD diagnosis had to be confirmed genetically and/ or by muscle biopsy. An additional criterium for the current study was that there was at least one FDP measurement available. Females were excluded as DMD mutations in females have a great variability in phenotype [11]. Furthermore, people with an intermediate phenotype (being able to walk 10 meters independently after 16 years of age) were excluded. The study was approved by the local medical ethical committee (no. 2019-5760).

### 2.2 Clinical parameters

From the DDD, the following data were retrieved for each visit: date of visit, age at time of visit, functional status, and the reported goniometric data of the upper extremity, including the FDP length. Functional status was assessed by the Brooke scale and reported the functional abilities of the upper extremities on a 6-point scale [12, 13]. The Vignos scale was used to categorize the functional abilities of the lower extremities on a 10-point scale [14, 15]. Disease stages were defined according to the guidelines developed by Bushby et al. [29]: the early ambulatory stage (EAS) (Vignos 1–3), the late ambulatory stage (LAS) (Vignos 4–8), the early non-ambulatory stage (ENAS) (Vignos 9–10, Brooke 1–3), and the late non-ambulatory stage (LNAS) (Vignos 9-10, Brooke > 4).

In both centers, the length of the long finger flexors, further referred to as FDP outcome, was determined by measuring the maximal passive wrist extension with the fingers fully extended using a manual goniometer and expressing FDP outcome in degrees [7]. Longitudinal analyses excluded the people who were not able to extent their fingers, because in that case, the FDP outcome could not be measured.

### 2.3 Statistical analysis

Statistical analysis was conducted using SPSS version 25.0 (IBM SPSS, Inc., Armonk, New York) and Stata/SE 16.0 for Windows (StataCorp LLC, Texas).

First, descriptive statistics were used to summarize enrollment characteristics at first visit. Means and standard deviations were used for continuous variables, frequencies (percentages) were used for categorical variables. Longitudinal graphs were used to depict the longitudinal course of the FDP outcome over time in different disease stages and to explore difference between the right and left hand.

Second, mixed model analyses were used according to the restricted maximum likelihood estimation procedure to quantify the FDP outcome over time, per disease stage and lastly, to determine the FDP outcome per year in each Brooke score. All mixed model analyses were corrected for age, and ‘age squared’ was used to correct for non-linear relation with age, if this was significant (*P* < 0.02).

## 3 RESULTS

### 3.1 Enrollment data and explorative longitudinal analyses

Data on 534 visits of 197 males were included, with on average 2.7 visits per male (range 1–5 visits). Patient characteristics and enrollment FDP outcome data of the total group and per disease stage are displayed in Table 1. The disease stage at enrollment could be defined for 146 males based on the available Brooke and Vignos scales. No significant differences in FDP outcome were found between the right and left hands. Cross sectional data in different disease stages showed a decrease in FDP outcome during disease progression. Furthermore, in the ambulatory disease stages and the early non-ambulatory disease stage, all people were able to passively fully extend the fingers. In the late non- ambulatory stage full passive extension of the finger joints was not possible in 18 (34%) of the right hands and 19 (31%) of the left hands. For this reason, FDP outcome was missing and were excluded from the longitudinal analysis.

**Table 1:** Characteristics and FDP outcome of the males with DMD at enrollment

	Total	EAS**	LAS	ENAS	LNAS
N	197*	38	12	34	62
Age (years), mean (range)	16.2 (4.0–48.3)	7.7 (4.0–14.5)	13.5 (8.9–23.0)	15.0 (8.9–23.1)	22.9 (11.4–48.3)
Full passive finger extension	168 (86)	38 (100)	12 (100)	34 (100)	41 (66)
Right hand Yes, N (%)
Full passive finger extension	172 (87)	38 (100)	12 (100)	34 (100)	43 (69)
Left hand Yes, N (%)
Mean FDP outcome*** right (degrees) (SD)	45.5 (38.4)	78.5 (12.5)	57.7 (32.2)	54.4 (24.7)	7.9 (38.6)
Mean FDP outcome left (degrees) (SD)	45.9 (38.9)	78.6 (11.2)	56.2 (30.1)	57.9 (22.1)	13.3 (40.0)

*The total number of patients which were measured. This number exceeded the sum of the patients in different disease stages as the Vignos and Brooke scales were missing for 51 patients. **EAS: Early ambulatory stage, LAS: Late ambulatory stage, ENAS: Early non-ambulatory stage, LNAS: Late non-ambulatory stage. ***FDP outcome: Indication of FDP length measured through wrist extension with extended fingers by goniometry.

**Fig. 1:**
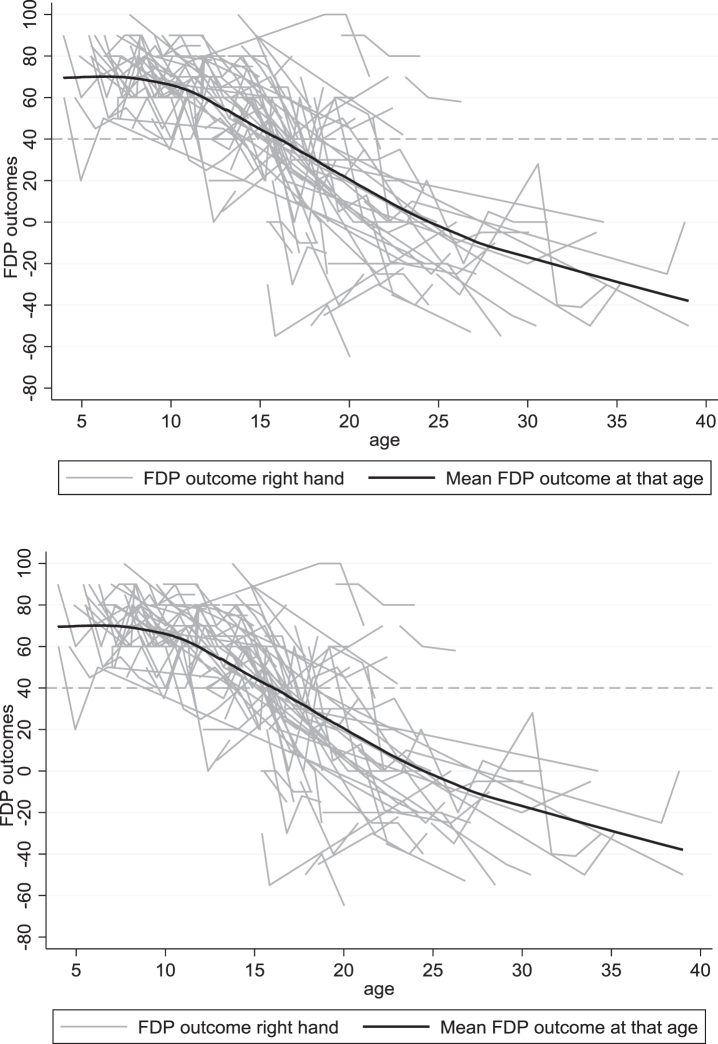
Longitudinal data of the right and left FDPs of the total group, set out with age.

**Fig. 2:**
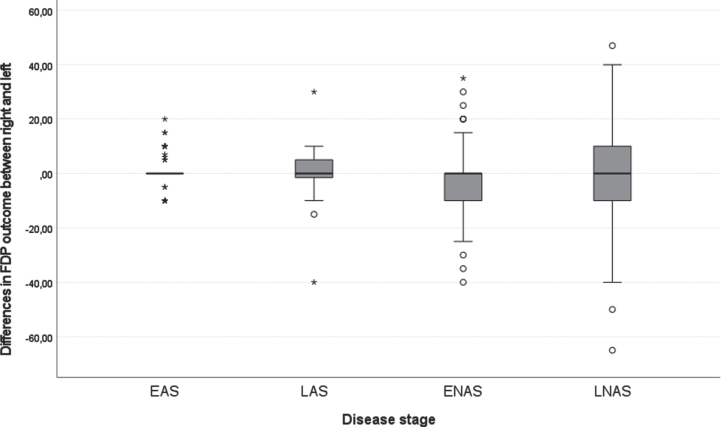
Boxplot of the differences of FDP outcome in the right and left hand of all longitudinal data in degrees for the different disease stages. Note: all longitudinal measurements are included, for this reason, a person may be represented in multiple boxplots as they may have changed from one disease stage to the next during the course of the study. EAS: Early ambulatory stage, LAS: Late ambulatory stage, ENAS: Early non-ambulatory stage, LNAS: Late non-ambulatory stage.

Figure 1 displays the longitudinal trajectories of FDP outcome per hand. Before the age of 10, this range is generally above 40 degrees, whereafter, there is a declining trend. The variation of FDP outcome between males enlarges with age. Figure 2 demonstrates the difference between the right and left FDP outcome per disease stage. The variation in differences between left and right increases with increasing disease stage with most variation in the late non-ambulatory stage. Still, the mean difference between the right and left hands is in around 0 in all disease stages.

**Fig. 3:**
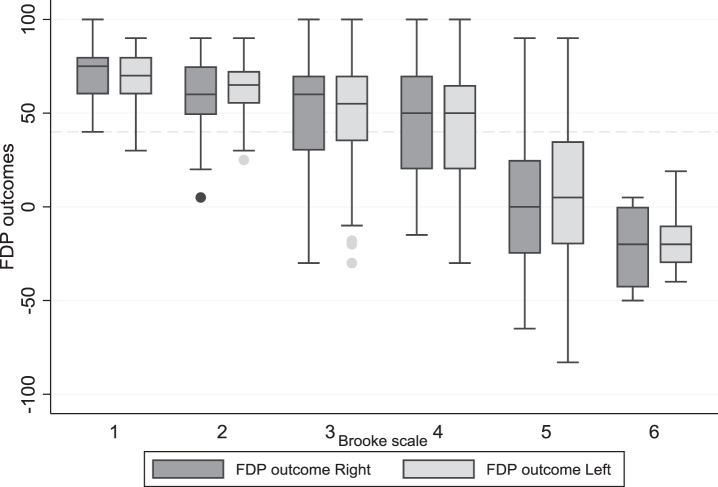
Boxplot of all longitudinal data of the FDP outcome left and right in the different Brooke scores (people who were not able to extend their fingers excluded). Note: all longitudinal measurements are included, for this reason, a person may be represented in multiple boxplots as they may have changed from one disease stage to the next during the course of the study.

In Fig. 3 the longitudinal data of both hands are set out in a box-plot for the different Brooke scale scores, which shows that with Brooke 1 and 2, only few FDP outcome are below 40 degrees. In Brooke 4, 41% of the measured FDP outcome in Brooke 4 are less than 40 degrees (for both sides). In Brooke 5 and 6, the majority of the FDP outcome is below 40 degrees.

### 3.2 Mixed model analyses

Mixed model analyses showed an overal β-estimate of –3.38 (*P* < 0.001) (right hand) and –3.57 (*P* < 0.001) (left hand) for age with the FDP outcome, which means that overall the FDPs tend to shorten around 3.5 degrees per year. As this relation is not expected to be exactly linear, we summarized the β estimates for the FDP outcomes for the right and left hand per disease stage (Table 2) and per year during each Brooke score (Table 3). Table 2 shows that when progressing from the early non-ambulatory stage to the late non- ambulatory stage, there is a significant decline in FDP outcome in both hands (resp. right/left: –11.4, –20.1 degrees, *P* < 0.001). Table 3 displays the difference in FDP outcome per year in the different Brooke scales. The biggest decline per year is seen in Brooke 5; –15.84 degrees (*P* < 0.001) for the right hand and –15.22 degrees (*P* = 0.002) for the left hand. Additional mixed model analyses showed a high correlation between the right and left FDP outcome (0.93, *P* < 0.001), indicating a symmetry in longitudinal decline. The correlation in the LAS is (0.88, *P* < 0.001).

**Table 2:** Mixed model analyses: FDP outcomes per disease stage, all disease stages are compared to the Early Ambulant Stage (EAS, constant). The regression is corrected for age. The beta estimate represents the mean difference in FDP outcome between the different disease stages. EAS is taken as constant

Right hand*	β estimates	P	(95% Conf. Interval)	Left hand**	β estimates	P	(95% Conf. Interval)
LAS	–1.91	0.582	(–8.78, 4.94)	LAS	–3.23	0.290	(–9.22, 2.77)
ENAS	–4.97	0.166	(–12.03, 2.09)	ENAS	–0.45	0.652	(–7.93, 4.99)
LNAS	–16.37	0.001***	(–25.79, –6.95)	LNAS	–20.59	0.006***	(–20.58, –3.49)
Age****	–3.38	<0.001***	(–4.03, –2.74)	Age	–3.57	<0.001***	(–4.18, –2.97)
Constant (EAS)	101.99	<0.001***	(94.11, 109.86)	Constant (EAS)	103.75	<0.001***	(95.99, 111.51)

*For the right hand: *n* = 160, total of 379 visits analyzed. **For the left hand: n=159, total of 380 visits analyzed. ***P <  0.01. ****Correction for the non-linear connection between FDP outcome and age (age^2^) was not significant and removed. EAS: Early ambulatory stage, LAS: Late ambulatory stage, ENAS: Early non-ambulatory stage, LNAS: Late non-ambulatory stage.

**Table 3:** Decline in decrease per year within each Brooke scale. The Beta estimate represents the change per year for the patients in that Brooke scale. Note: all visits are included. A person may have changed from one disease stage to the next during the course of the study and can therefore be counted in more than one disease stage

Right hand	β estimates	P	(95% Conf. Interval)	Left hand	β estimates	P	(95% Conf. Interval)
Age_Br* 1	–0.95	0.055	(–1.93, 0.26)	Age_Br 1	–1.34	0.019	(–2.42, –0.25)
Age_Br 2	–1.49	0.113	(–3.40, 0.42)	Age_Br 2	–5.91	0.243	(–17.3, 5.48)
Age_Br 3	–2.00	0.058	(–4.02, 0.07)	Age_Br 3	–2.65	0.007**	(–4.49, –0.77)
Age_Br 4	–2.94	0.196	(–8.12, 2.25)	Age_Br 4	–3.96	0.228	(–10.61, –2.69)
Age_Br 5	–15.84	<0.001**	(–21.85, –9.83)	Age_Br 5	–15.22	0.002**	(–21.86, –8.59)
Age_ Br 6	–0.49	0.725	(–3.21, 2.37)	Age_Br 6	0.59	0.335	(–0.79, 1.96)

*Age_Br=Beta estimate represents the change per year for the patients in that Brooke scale. Number of included patients/ visits per Brooke scale: Brooke 1 R: N = 51, 101 visits L: N = 51, 101 visits. Brooke 2 R: N = 41, 56 visits, L: N = 56, 41 visits. Brooke 3 R: N = 49, 84 visits, L: N = 84, 49 visits. Brooke 4 R: N = 22, 26 visits, L: N = 26, 22 visits. Brooke 5 R: N = 57, 105 visits L: N = 56, 105 visits. Brooke 6: R: N = 10, 16 visits, L: N = 10, 17 visits. **P <  0.01.

## 4 DISCUSSION

This is the first longitudinal study which describes in detail the course of the long finger flexors length decline in patients with DMD. Results show that the decline in FDP outcome is largely symmetrical, however in the late non-ambulatory disease stage more variability occurs between the two hands. Decline of the length of the long finger flexors is largest during the Brooke 5 score (resp. right/ left: –15.8, –15.2 degrees per year). During the Brooke 4 disease stage, however, already 41% of the measured FDP outcome was below 40 degrees which is generally acccepted as a threshold in being able to elaborate manual precision tasks [8].

The longitudinal course of the FDP outcome shows that for the majority of the people with DMD, FDP shortening is not an issue until the age of 10, which is in line with previous cross-sectional findings by McDonald et al. [16] and with the longitudinal MRI abnormalities in the FDPs, investigated by Brogna et al. [17]. However, hand weakness already exists at a young age [18] and also limitations in activity of the upper extremity was found in young people with DMD before [3, 19]; both can limit the active use of the full range of motion and lead to shortening of muscles in the longer term. Active use of the upper limbs in the early stages of DMD, especially the extension of wrist and fingers may delay the shortening of the FDPs.

After the age of 10, a decline in FDP outcome is seen in both hands, which is significantly correlated with age and functional status. Preventive measures, such as stretching and wearing orthoses should be started before the hand function declines. As almost half of the FDP outcome was below 40 degrees in Brooke 4, we recommend to start preventive measures before transitioning to this stage. Moreover, attention is needed for people with DMD within the Brooke 5 as the FDP outcomes decline very rapidly. However, it is seen that in the later disease stages the variability increases, which means that interventions need to be personalized according to the annual follow-up of the FDP outcome in combination with the Brooke score.

The majority of the FDP outcomes is symmetrical i.e., no significant differences were seen between the right and left hand in enrollment data, a high correlation existed between the two sides in longitudinal analyses, and the mean difference between the FDP outcomes between the right and left hands was zero in all disease stages. Symmetric decline in the upper limbs has been seen before in the study of Janssen et al. [3]. However, for some participants a difference did exist up to 40 degrees, which confirms that personalized care is important. Future research on preventive measures can benefit from the largely symmetrical decline, as it is possible to use the contralateral side as a control.

The FDP outcome could not be measured in all cases in the late non-ambulatory stage, due to the inability to passively fully extend the fingers due to contractures in the metacarpal and/ or finger joints. Other causes for missing values were that measurements were too painful. This has to be taken into account when interpreting these results. Hopefully, preventive measures can reduce pain and limitations in finger mobility in the late non ambulatory disease stage in the future.

Hand function is influenced by FDP contractures as wrist extension is important in the conduction of fine motor tasks [8], and also the grip strength is higher with wrist extension compared to wrist flexion [20]. With already existing hand weakness, it is even more important to maintain wrist extension range of motion. The maintenance of hand function and the ability to conduct activities with the upper extremity may make a large difference to be able to participate in different life roles. Janssen et al. [21], found associations between upper extremity function and living an active life by participating in school and work-related activities. Timely interventions, such as prevention of FDP contractures, but also support of active use of the upper extremity, including wrist- and finger extension, may enhance participation, which is increasingly important in the context of the longer life expectancy of people with DMD [22].

This study needs to be interpretated in the light of its strengths and weaknesses. The strengths of this study are that we were able to longitudinally investigate FDP outcome in a large number of patients. Second, the results were interpreted after a correction for age and age square, which means that results can be interpreted in the light of the disease stages, independent from age and linearity was taken into account. The weaknesses of this study included the retrospective design which always includes missing data and the possibility of entry errors. Precise data analysis and the large study population allows the interpretation of the data to still be very valuable. Second, data on corticosteroid use and on preventive measures such as stretching, wearing orthosis, and positioning of the hands were not included, as information on dosage and compliance was lacking. Despite the fact that this additional information could possibly have differentiated the population into subgroups, the results of the present study still provide a good impression of the overall course of long finger flexor length, irrespective of treatment modalities. Prospective analyses of the FDP outcome in people with DMD wearing and not-wearing orthoses would be very useful in future research. Third, only few people were in the late ambulatory stage, which is probably because this stage is often very short, and in the Netherlands, the use of long leg braces is scarce. At last, the measurement of the FDP outcome is not yet validated and measurement errors can exist [23]. Although, both centers have a dedicated neuromuscular team, which is involved in these measurements for 6 years now, we need further research into the reliability of this measurement.

In conclusion, our retrospective exploration of the FDP decline, showed that in our DMD population the largest decline occurred within Brooke score 5. In persons with Brooke score 4, already 41% of the FDP outcomes were below 40 degrees. We recommend to consider preventive measures from Brooke score 4 onwards for persons with DMD who show a decline in FDP length.. In addition, this article highlights the need for a prospective study of FDP outcomes, including data on preventive measures, corticosteroid use and functional outcome measures, to improve understanding of contracture prevention.
